# *Sonerilacardamomensis* (Melastomataceae), a new species from Cambodia

**DOI:** 10.3897/phytokeys.156.55866

**Published:** 2020-08-21

**Authors:** Jae-Seo Shin, Bo-Kyeong Song, Chhang Phourin, Hyosig Won, Kyong-Eun Lee, Seong-Hyun Cho

**Affiliations:** 1 Department of Life Science, Hallym University, 1 Hallymdaehak-gil, Chuncheon-si, Gangwon 24252, South Korea Hallym University Chuncheon South Korea; 2 Forestry Administration, 40 Preah Norodom Blvd, Phnom Penh, Kingdom of Cambodia Forestry Administration Phnom Penh Cambodia; 3 Department of Biological Science, Daegu University, Gyeongsan 38453, South Korea Daegu University Gyungsan South Korea; 4 National Institute of Biological Resources, Incheon 22689, South Korea National Institute of Biological Resources Incheon South Korea

**Keywords:** Cambodia, Central Cardamom Protected Area, new species, *Sonerila*

## Abstract

*Sonerilacardamomensis*, a new species of family Melastomataceae from the Central Cardamom Protected Area in Koh Kong province of southwestern Cambodia, is described and illustrated. The species is similar to *S.violifolia* Hook.f. ex Triana, but is readily distinguished by its cordate leaf base, lack of setae at the nodes, longer pedicels, smaller petals, smaller hypanthiums and smaller capsules.

## Introduction

Melastomataceae Juss. consists of approximately 166 genera and 4,200–4,500 species which are distributed in both the New World (about 2,950 species) and the Old World (1,275–1,550 species) (Renner 1993, Michelangeli et al. 2013). In Cambodia, Melastomataceae are currently represented by 34 species and 6 varieties for a total of 40 taxa in 9 genera (Cho et al. 2015, Tagane et al. 2015, Cho et al. 2016a, Cho et al. 2016b). The most species-rich genera in Cambodia are Memecylon L. (18 species), Osbeckia L. (7 species), and Melastoma L. (5 species).

In Indochina, there are around 22 species of *Sonerila* and the present count includes 9 species from Vietnam, 13 species from Thailand and 4 from Laos (Guillaumin 1913, Ho 1999, Renner et al. 2001, Chen and Renner 2007, Newman et al. 2007). In Cambodia, at the beginning of the 20^th^ century, only one species was described, namely *Sonerilaquadrangularis* (synonym of *S.maculata* Roxb.). Three species are reported in the present account, namely *S.bokorense* S.H. Cho & Y.D. Kim, *S.maculata* Roxb., and *S.plagiocardia* Diels (Cho et al. 2016a).

**Figure 1.**
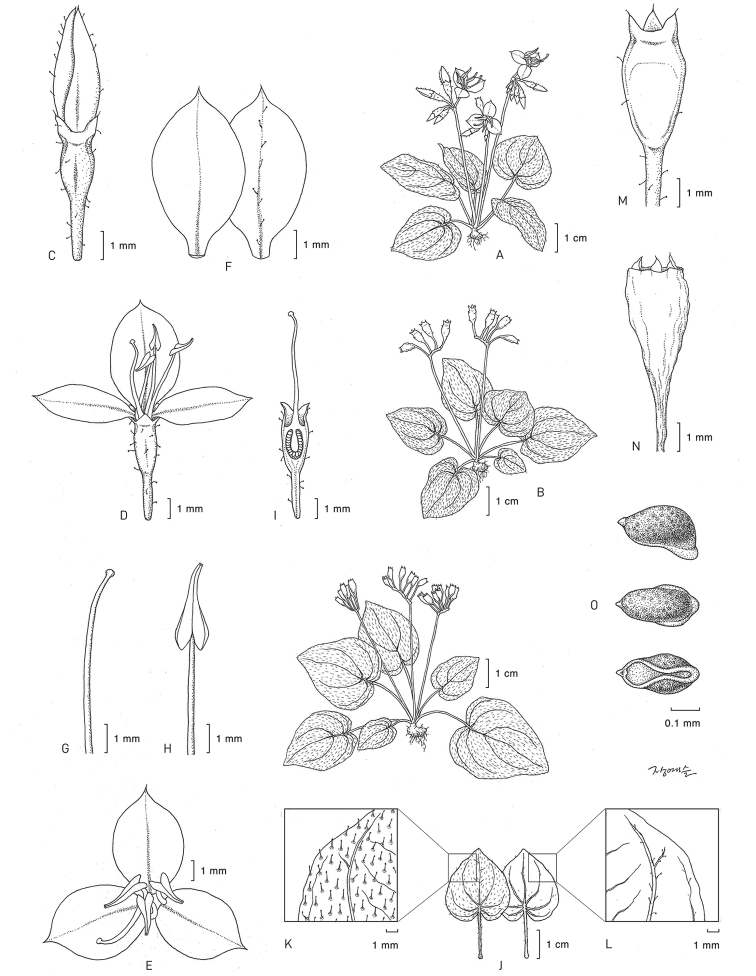
*Sonerilacardamomensis***A** Flowering individual **B** fruiting individual **C–D** developing flower **E** mature flower **F** petals (right: abaxial, left: adaxial) **G** style and Stigma **H** filament and Anther **I** gynoecium **J** leaf **K** upper surface of leaf **L** lower surface of leaf **M** immature capsule **N** mature capsule **O** seeds: Cho et al. CB-4467. Illustration by Ye-Seul Jang.

**Figure 2.**
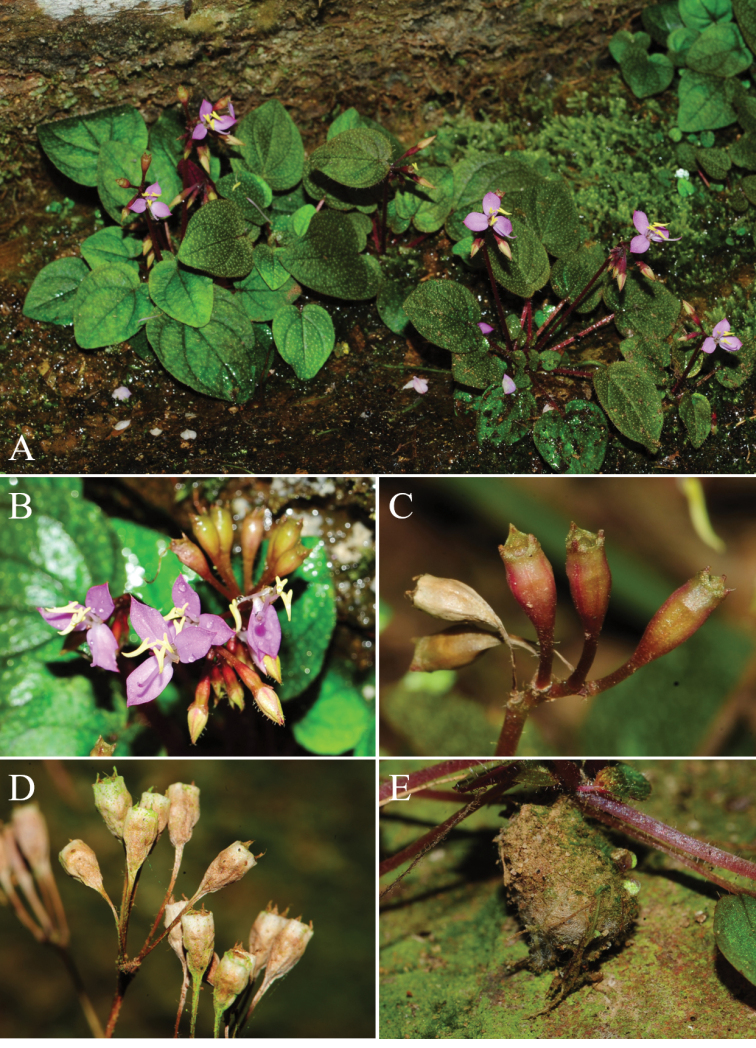
**A–E***Sonerilacardamomensis***A** habit **B** flowers **C** immature capsules **D** mature capsules **E** short bulbous rhizome: Photos by Seong-Hyun Cho.

During the recent floristic survey, one species of *Sonerila* was collected at Thmor Bang District of the Central Cardamom Protected Area in Koh Kong province of southwestern Cambodia that does not appear to be similar to previously described species (Fig. 1, 2). It is the most similar to *Sonerilaviolifolia* Hook.f. ex Triana, but detailed examination of the morphology revealed that it differs from *S.violifolia*. Therefore, it is described here as a new species.

## Taxonomy

### Sonerila cardamomensis

Taxon classificationAnimaliaMyrtalesMelastomataceae

S.H.Cho sp. nov.

3CE7F5C7-0D19-5C24-94D4-E18CA646DBC2

urn:lsid:ipni.org:names:77211172-1

Figures 1 , 2

#### Type.

Cambodia. Koh Kong Province, Central Cardamom Protected Area, sandstone rocky area in evergreen forest, 11°42'08.0"N, 103°26'22.5"E, a.s.l. 427 m, 9 August 2018, with flowers, Cho S.H., Shin J.S., Song B.K., Chhang Phourin CB-4467 (holotype KB!, isotypes KB!, K!, P!).

#### Diagnosis.

*Sonerilacardamomensis* is most similar to *S.violifolia* Hook.f. ex Triana, which is distributed in Myanmar and Thailand but is readily distinguished from the latter by the cordate leaf base, lack of setae at the nodes, longer pedicels, smaller petals, smaller hypanthia and smaller capsules (Table 1).

**Table 1.** Comparison of key features of *Sonerilacardamomensis* and *S.violifolia*.

Taxonomic traits	*Sonerilacardamomensis*	*S.violifolia*
Stem setae at the nodes	Absent	reddish brown, 4.0–6.0 mm long
Leaf upper surface base	glandular trichomes cordate	spares minute bristly hairs obtuse or cuneate
Pedicel	ca.4.0 mm long	2.0–3.0 mm long
Hypanthium	ca. 3.5 mm long	5.0–6.0 mm long
Petal	5.5–6.0 mm long	8.0–10.0 mm long
Capsule	3.5–4.0 mm long	6.0–7.0 mm long

#### Description.

Perennial, herbs, terrestrial, 4–7 cm high, 1 (or rarely 2) shoot from rhizome. Rhizome short, bulbous, 0.4–1.0 cm in diam. Stems 4-sided, tinged purplish, the internodes almost absent. Leaves simple, membranous, opposite, clustered at base, those of a pair isomorphic; petioles 0.4–2.5 cm, glandular trichome; leaf blade ovate, 1.0–4.0 × 0.9–2.7 cm, apex acute, rarely obtuse, base cordate, upper surface covered with glandular trichome, lower surface slightly covered with glandular trichome, lateral primary vein 2–3 pairs, departing at the base, margin serrate. Inflorescences pedunculate, 1–5[7] scorpioid cymes with 5 to 11 flowers; peduncle [1.5]3.0–5.0 cm (up to 8.0 cm long when fruiting), angular with glandular trichome. Pedicel ca. 4.0 mm long (up to 5.0 mm long when fruiting), with glandular trichomes. Flowers bisexual, 3-merous. Hypanthium campanulate, 3-angled, 6-ribbed, 3.5 × 1.3–1.5 mm, with glandular trichomes. Calyx lobes broadly triangular 0.7 × 1.0 mm, apex acute. Petals 3, thin, 5.5–6.0 × 3.5–4.0 mm, ovate-obovate, apex acute to acuminate, pink to purplish pink, abaxially glandular trichome on midveins, adaxially glabrous. Stamens 3, isomorphic; filaments distinct, 3.7–4.0 mm, glabrous, pinkish; anthers ca. 4.5 mm, lanceolate, deeply cordate to sagittate at the base, apex acute, yellow, dehiscence poricidal. Ovary inferior, urceolate, apex with a membranous 3-lobed crown, ovules numerous, placentation axillary; style filiform, 6.6–7.3 mm, glabrous, pink; stigma apiculate. Fruit a capsule, campanulate, 3.5–4.0 × 2.0–2.2 mm, 6 longitudinal ribs, glabrous. Seeds cuneate, numerous, ca. 0.3 mm, light brown.

#### Specimen examined.

Cambodia. 9 August 2018, with flowers, Cho et al. CB-4469, 4471, 4473 (KB!), Won et al. 16499, 16506(DGU!); 15 January 2019, with fruits, Cho et al. CB-4614, 4615 (KB!)

#### Phenology.

Fls July to August; Frts August to November.

#### Distribution and habitat.

*Sonerilacardamomensis* grows on sandstone rocky area in evergreen forest from 420 to 600 m.a.s.l. Endemic to southwestern Cambodia, *S.cardamomensis* is at present known only in the Central Cardamom Protected Area in Koh Kong province.

#### Conservation status.

*Sonerilacardamomensis* was collected in the Central Cardamom Protected Area of Koh Kong province in southwestern Cambodia. Until now, two big populations are known, each composed of more than ca. 1,000 individuals, and was discovered in the protected area. Therefore, it is preliminarily classified as data deficient (DD) according to the IUCN Red List criteria.

## Supplementary Material

XML Treatment for Sonerila cardamomensis
